# Do Pre‐Clinical Summative Assessments Predict a Student's Clinical Performance? A Retrospective Study

**DOI:** 10.1111/eje.13098

**Published:** 2025-05-07

**Authors:** Yasmina Andreani, Buddhi Champika Gunaratne, Atieh Sadr, Fjelda Elizabeth Martin, Tihana Divnic‐Resnik, Smitha Sukumar

**Affiliations:** ^1^ Sydney Dental School Faculty of Medicine and Health, the University of Sydney Sydney Australia; ^2^ UWA Dental School University of Western Australia Nedlands Australia

**Keywords:** assessment, clinical skills, dental education, pre‐clinical, simulation, summative

## Abstract

**Introduction:**

Dental students are deemed fit to treat patients (clinical readiness) based on their performance in pre‐clinical summative assessments. This involves assessing knowledge (theory exams) and technical skills (simulation‐based activities). However, there is weak evidence to support whether these pre‐clinical assessments accurately predict clinical performance. The aim of this study was to determine if pre‐clinical summative assessments predicted the clinical performance of students in a graduate dental programme.

**Materials and Methods:**

This retrospective longitudinal cohort study analysed the results of pre‐clinical (theory, simulation) and clinical summative assessments in Restorative Dentistry, Periodontics and Endodontics from six cohorts of second‐ and third‐year students (2013 to 2019) enrolled in The University of Sydney's Doctor of Dental Medicine program. The association between pre‐clinical (theory and simulation) marks with clinical marks were analysed by discipline using Pearson's correlation coefficient (*r* ^2^).

**Results:**

A weak but significant positive correlation was found between a student's pre‐clinical theory mark and their clinical performance in all three disciplines. The only significant positive correlation between pre‐clinical simulation marks and clinical performance was found in Restorative Dentistry.

**Discussion:**

While some positive correlations were found between pre‐clinical and clinical performance, overall, these results indicate that pre‐clinical ability was not a reliable predictor of a student's clinical competence.

**Conclusions:**

Assessing clinical readiness is complex. Our results indicate this attribute may potentially be better assessed using a range of qualitative and quantitative metrics. Further research is required to better define and quantify clinical readiness.

**Abbreviations**
DMD: Doctor of Dental Medicine
Endo: endodontics
OSCE: objective structured clinical examination
Perio: periodontics
SAQ: short answer question SBA: single best answer

## 1 Introduction

Dental schools worldwide run comprehensive pre‐clinical programs consisting of didactic and simulation learning in different disciplines to prepare students for patient clinics [1]. While each discipline requires the acquisition of a specific set of psychomotor skills, the underlying pedagogical framework is based on repetitive learning [2]. This means students have multiple attempts to develop fine motor skills following a specified protocol using plastic teeth for a standardised learning experience in a safe environment. Pre‐clinical simulation training activities are integrated with a didactic program designed to build knowledge in both discipline‐specific subjects and broader areas such as life sciences, dental materials science, communication skills, professionalism and ethics [1]. This hierarchical education model operates on the principle that developing technical skills and foundational knowledge during pre‐clinical years prepares students for patient clinics [3]. Notably, it assumes that success in pre‐clinical assessments directly correlates with clinical readiness, ensuring the safe and effective management of patients.

The evidence to date regarding the prognostic ability of pre‐clinical theory (didactic) and practical (simulation) assessments is limited and inconclusive. The literature pertaining to theory assessments has primarily focused on Objective Structured Clinical Examinations (OSCE) which are used in the clinical years [4, 5]. This type of exam may not be relevant in the context of dental pre‐clinical training. Instead, Single Best Answer (SBA) [6] and Short Answer Questions (SAQs) are more commonly used for assessing foundational knowledge and theoretical understanding of technical skills.

In dentistry, a limited number of studies have examined the relationship between pre‐clinical theory and simulation marks [7, 8, 9], rather than the connection between theoretical knowledge and clinical performance in the patient clinic. The results provide no clear consensus that didactic marks can predict simulation performance. While two studies found no correlation [7, 8], another identified a weak but significant positive correlation between theoretical knowledge in dental anatomy and simulation performance in restorative dentistry [9].

The literature also provides conflicting data regarding the usefulness of pre‐clinical simulation performance in reliably predicting clinical performance. In the simulation setting, studies analysing marks from multiple disciplines showed that the ability of students to complete procedures on plastic teeth could not predict later clinical performance [10, 11]. However, other studies showed a weak positive correlation between discipline‐related pre‐clinical and clinical performance [12, 13]. Endodontics studies rarely make direct comparisons between students' simulation and clinical performance [14]. Instead, the focus is on evaluating the success of endodontic procedures (RCT) performed on extracted versus plastic teeth. Results from these studies indicated that a student's performance was not significantly different based on the type of tooth used [15, 16]. Nevertheless, these outcomes must be interpreted with caution as the prevailing education paradigm indicates that plastic teeth cannot fully replace extracted teeth in the endodontic simulation setting [17].

Overall, the findings to date suggest that the relationship between pre‐clinical and clinical performance is not fully understood. This is largely due to methodological differences such as the lack of standardisation of study populations (clinicians with varying levels of experience were assessed), significant variation between when pre‐clinical and clinical assessments are completed, and the inclusion of clinical assessments which integrated one or more disciplines [10, 12, 13, 18].

Notably, there is a paucity of data regarding pre‐clinical and clinical performance in periodontics, a fundamental aspect of general dental training.

Given the identified gaps and lack of evidence using real‐world educational outcomes, this retrospective study examined whether pre‐clinical assessments (simulation and theory) could predict students' clinical performance in three core disciplines restorative dentistry (Restorative), periodontics (Perio) and endodontics (Endo) in the 4‐year Doctor of Dental Medicine programme at the University of Sydney. These disciplines were specifically chosen as they are universally recognised as core areas of general dentistry and are taught throughout the curriculum, which enabled longitudinal analysis.

## 2 Materials and Methods

Study population The study used a non‐randomised retrospective sample of second‐ and third‐year dental students enrolled at Sydney Dental School between 2013 and 2019, whose assessment results were collated for analysis. The study protocol was approved by the University Ethics Committee, protocol number (2021/337).

The total number of students in the six cohorts = 493. These cohorts were utilised for analysis as the class sizes were similar (median class size = 82 ± 8 [71–92]), see Table 1. Therefore, the simulation and clinical exposure were similar for all students included in this study (Tables S1, S2). Nine students were excluded from analysis as they were missing marks in more than one discipline over one or both years. Therefore, a total of 484 students were included in this study (Table 1). To avoid bias, repeating students were only included once with marks taken from their repeated year. No pre‐clinical theory marks were archived for cohort A (2013), therefore, only cohorts B (2014) to F (2018) were used for theory/clinic analysis (*n* = 413). The discrepancy between class size and number of students analysed in Cohorts B to F was due to the exclusion of students who (i) were removed from analysis due to incomplete marks in one or more categories over one or both years or (ii) failed to complete an Endodontic clinical case in DMD3 (*n* = 42). Endodontic clinic marks only include the students who completed their endodontic case, resulting in a smaller sample size (*n =* 442).

**TABLE 1:** Description of doctor of dental medicine (DMD) cohorts included in this study.

Cohort					Restorative dentistry		Periodontics		Endodontics	
	Class size	% female	DMD 2	DMD 3	Sim^a^ & theory	Clinic	Sim & theory	Clinic	Sim & theory	Clinic
A	71	43	2013	2014	71	71	71	71	71	69
B	77	39	2014	2015	76	76	76	76	76	74
C	82	51	2015	2016	81	81	81	81	81	78
D	92	51	2016	2017	89	89	89	89	89	67
E	90	47	2017	2018	87	87	87	87	87	81
F	81	41	2018	2019	80	80	80	80	80	73
Mean ± SD^b^	82 ± 8	46 ± 5			81 ± 7	81 ± 7	81 ± 7	81 ± 7	81 ± 7	74 ± 5
Median	82	45			81	81	81	81	81	74
Total	493				484	484	484	484	484	442

^a^ Simulation.

^b^ Standard deviation.

Data from the year 2020 onwards was excluded due to statewide Covid lockdowns through 2020–2021 in New South Wales, Australia which resulted in limited simulation and clinical exposure. All data was de‐identified for this analysis.

### 2.1 Doctor of Dental Medicine: Entry Requirements

Entry into this program requires a Grade Point Average (GPA) of 4.5 or 5 (7‐point scale) for rural and metropolitan/international applicants respectively. The candidates must pass a national, independently run entrance exam—Graduate Medical School Admission Test which—confirms a level of basic science knowledge as a prerequisite for all successful applicants. Candidates must attain a specific score, which is set by the university's admission centre annually. Having attained these requirements, candidates are invited to be interviewed through a Multiple Mini Interview (MMI) process. This is a calibrated and validated assessment process used to identify potential candidates entering dentistry and medicine [19].

### 2.2 Doctor of Dental Medicine: Structure

The DMD degree is a fixed diet programme with progression decided at the end of the academic year based on the assessments undertaken in semester 1 and 2. The programme consists of seven units of study—three foundational/pre‐clinical units which run over first and second year and three integrated clinical units which run in third and fourth year. Additionally, a research unit runs from DMD 1 to the end of semester 1 in DMD 4. Each unit of study is multi‐specialty/disciplinary and the subjects for each unit (Table S3). The units of study run concurrently; therefore, student learning in the pre‐clinical simulation programme occurs in the context of gaining knowledge across a diverse range of subjects from basic life sciences, including anatomy, as well as tooth morphology and communication. The pre‐clinical simulation programme starts in the first year of the degree and spans two‐and‐a‐half years. Plastic teeth are used in simulation training exercises across all clinical disciplines. However, extracted teeth were also used in Restorative for caries removal and in Endo exercises for access cavity preparation, canal preparation and obturation.

Students have introductory clinical courses in history taking, basic examination skills, cariology and Perio in DMD 1 semester 2 and DMD 2 semester 1 using peers as patients. Students are credentialled healthcare workers of the state government health service (NSW Health) and see patients eligible for public dental care in accredited clinical facilities affiliated with the University of Sydney from DMD 2 semester 2 to the end of DMD 4. The clinical sessions in DMD 2 semester 2 are limited to history taking and examination, plus providing patients with a scale and clean. This runs concurrently with the periodontics and endodontics simulation programmes. Sessional assessment (feedback and procedural marks) in this introductory course is formative and has not been included in this study.

In DMD 3 and 4 the focus is on establishing and expanding the students' clinical skills. The units of study align with broader university administrative structures that do not reflect patient care. The students work in integrated clinics completing Restorative, Endo and Prosthodontics under generalist and specialist supervision. Specialist clinics are run for Perio (DMD 3 and 4), exodontia (DMD 3 and 4), Paediatric Dentistry, Orthodontics and Special Care (DMD 4). Clinic sessions increase from one clinic session a week in Semester 2 in DMD 2 to an average of two to three clinical sessions/week for Restorative including Endo in DMD 3. Additionally, students treat patients in specialist Perio clinics regularly throughout DMD 3. In DMD 4 students treat patients eight sessions/week over 32 weeks. This is significantly longer than the standard Australian university academic year of 26 weeks.

### 2.3 Data Collation and Calibration for Summative Assessments

**FIGURE 1:**
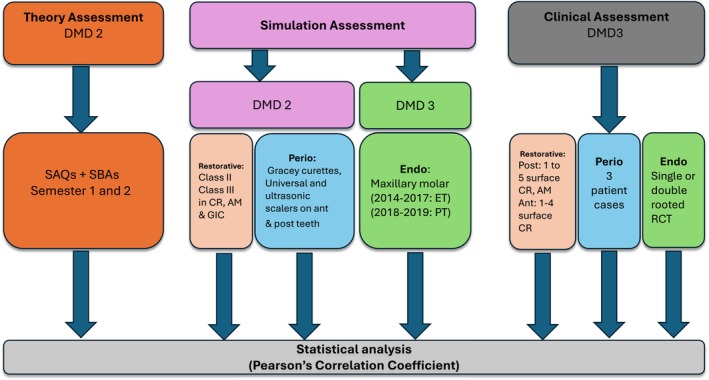
Flow diagram outlining study methodology. All marks were taken from the second and third year of the Doctor of Dental Medicine (DMD) program from Restorative Dentistry (Restorative), Perio (Periodontics) and Endo (Endodontics). Theory and simulation assessments comprise the pre‐clinical component, which was compared to students' clinical assessment based on specific procedures. Simulation assessments were undertaken on plastic teeth (PT) in Restorative and Perio, while extracted teeth (ET) were used in Endo between 2014 and 2017. Simulation assessment in Restorative required students to complete restorations in resin composite (CR), amalgam (AM), and glass ionomer cement (GIC). Perio simulation procedures were undertaken in anterior (ant) and posterior (post) teeth.

This study is a retrospective cohort analysis of summative assessment marks in pre‐clinical theory, simulation and clinical assessments for three disciplines. Data collation from pre‐clinical and clinical assessments are summarised in Figure 1.

Calibration of the teaching staff is a prerequisite for the delivery of high‐quality dental education. Therefore, significant effort was made at Sydney Dental School to ensure standardised assessment processes across all pre‐clinical and clinical assessments. This included a standardised format for theory papers, rubric‐driven assessment for simulation assessments and a clinical rubric. Variations in standardisation are mainly due to discipline‐specific requirements (each disciple has its own rubrics) but the calibration process underpinning all three disciplines was the same. Moreover, there was constant evaluation of methods used across the three disciplines over the seven‐year period the data was collected. All authors were involved in assessments in the period analysed and worked together to embed standardisation in all aspects of teaching and assessments. The main variation among the assessments during the study period was a change in the type of teeth used in the Endo simulation assessments, moving from extracted to plastic teeth in 2018. A subgroup analysis was undertaken to identify if this impacted student performance in clinics.

### 2.4 Theory

The theory mark is a single percentage which represents student performance in written papers undertaken at the end of each semester in DMD 2. As each discipline is part of a multi‐speciality unit of study, students sit a 2‐h assessment each semester covering a range of subjects; for example, Restorative and Endo are examined in the same paper. We extracted marks from discipline‐specific questions (20 SBA's and 10 SAQ's) within each paper (Figure 1). Sydney Dental School maintains a secure exam question repository which is regularly curated by a select group of trained and calibrated content specialists. All questions are reviewed by respective heads of discipline. Model answers are provided for all SAQ's to facilitate standardised marking. Failed questions are reviewed and reassessed by an independent marker.

### 2.5 Simulation

The marks are taken from assessments completed at the end of DMD2 Semester 2 for Restorative and Perio. For Endo, DMD3 Semester 1 marks were used. Cohorts A–D completed their simulation assessment on extracted teeth, while E and F used plastic teeth.

A small number of experienced examiners were involved in simulation assessments (10 in Restorative, two in Perio and Endo). They followed discipline‐specific rubrics and were calibrated with the opportunity to discuss discrepancies to ensure standardisation. Simulation assessments were always double‐marked to eliminate potential bias of individual tutors.

### 2.6 Clinics

The clinical marks collated were restricted to discipline‐specific procedures undertaken on patients in DMD 3 (see Figure 1). The clinical requirement in Endo in DMD 3 involved completion of a single or double‐rooted RCT for a patient. Students who did not complete their endodontic clinical requirement (*n* = 42) were excluded from the analysis.

A larger group of educators (approximately 30) was involved in clinical assessments over the 6 years. Students were assessed in every session, and each session contributed to their overall clinical mark based on the clinical rubric developed by a team of academics. This rubric was used by all educators. Generalists and specialists were trained and calibrated to use this rubric by the heads of disciplines (all authors of this paper) as part of a Clinical Educators Day run every January (beginning of the academic year).

This study used the marks for specific clinical procedures (Table S4) which were relevant to each discipline. In DMD 3 the real time grades for each procedure are categorical as defined by the rubric (unsatisfactory to proficient). These grades were collated by a group of eight highly experienced clinical educators/academics (including authors of this paper) and were then converted into a percentage. This highly calibrated process occurred at the end of each semester to ensure standardisation of clinical marks to counter the inherent inconsistencies expected when a large group of clinicians is involved in assessment.

### 2.7 Statistical Analysis

The association between theory and clinical, and simulation and clinical marks were analysed by discipline using Pearson's correlation coefficient (*r* ^3^) using the ggpubr package (v 0.6.0). Pearson's correlation coefficient is a number between −1 and 1. In this study, a positive number indicates a positive correlation, i.e., the pre‐clinical mark (theory or simulation) correlates with a clinical mark. This means the pre‐clinical mark is predictive of clinical performance. The strength of this correlation is designated by the *r* ^2^ value and can be categorised as strong (> 0.7 and < −0.7); moderate (0.4–0.6 and −0.4 to −0.) and weak (0–0.3 and 0 to −0.3) [20]. The raw data used for this analysis can be found in Table S5.

A subset correlation analysis was done within Endo as extracted teeth were used for the summative simulation assessments in cohorts A–D with the transition to plastic teeth for cohorts E and F.

All analyses were undertaken in r Studio (v4.2.2).

## 3 Results

This study analysed the summative results of 484 DMD students from six cohorts enrolled in DMD 2 and 3 between 2013 and 2019. The summary statistics for the marks analysed are presented in Table 2.

**TABLE 2:** Summary statistics of pre‐clinical and clinical marks analysed by discipline.

	*n* ^a^	Mean	SD	Median	Min	Max	Range	Skew	Kurtosis
Theory									
Restorative dentistry	413	70.7	11.6	72.7	34.3	92.7	58.4	−0.6	1.1
Endodontics	413	65.5	10.7	66.3	33.1	96.9	63.8	−0.2	−0.2
Simulation									
Restorative dentistry	484	63.0	11.2	62.9	0	89	89	−1.1	6.6
Periodontics	484	79.5	14.0	82.2	0	100	100	−1.8	7.0
Endodontics	484	69.6	12.5	71.1	16.7	96	79.33	−0.8	0.8
Clinic									
Restorative dentistry	484	52.8	10.4	49.3	35.7	90	54.3	1.4	1.9
Perio	484	54.1	12.2	49.4	34.8	90	55.2	1.1	0.4
Endo	442	54.4	12.1	49.5	0	90	90	1.2	3.4

^a^ Total number of students analysed.

**FIGURE 2:**
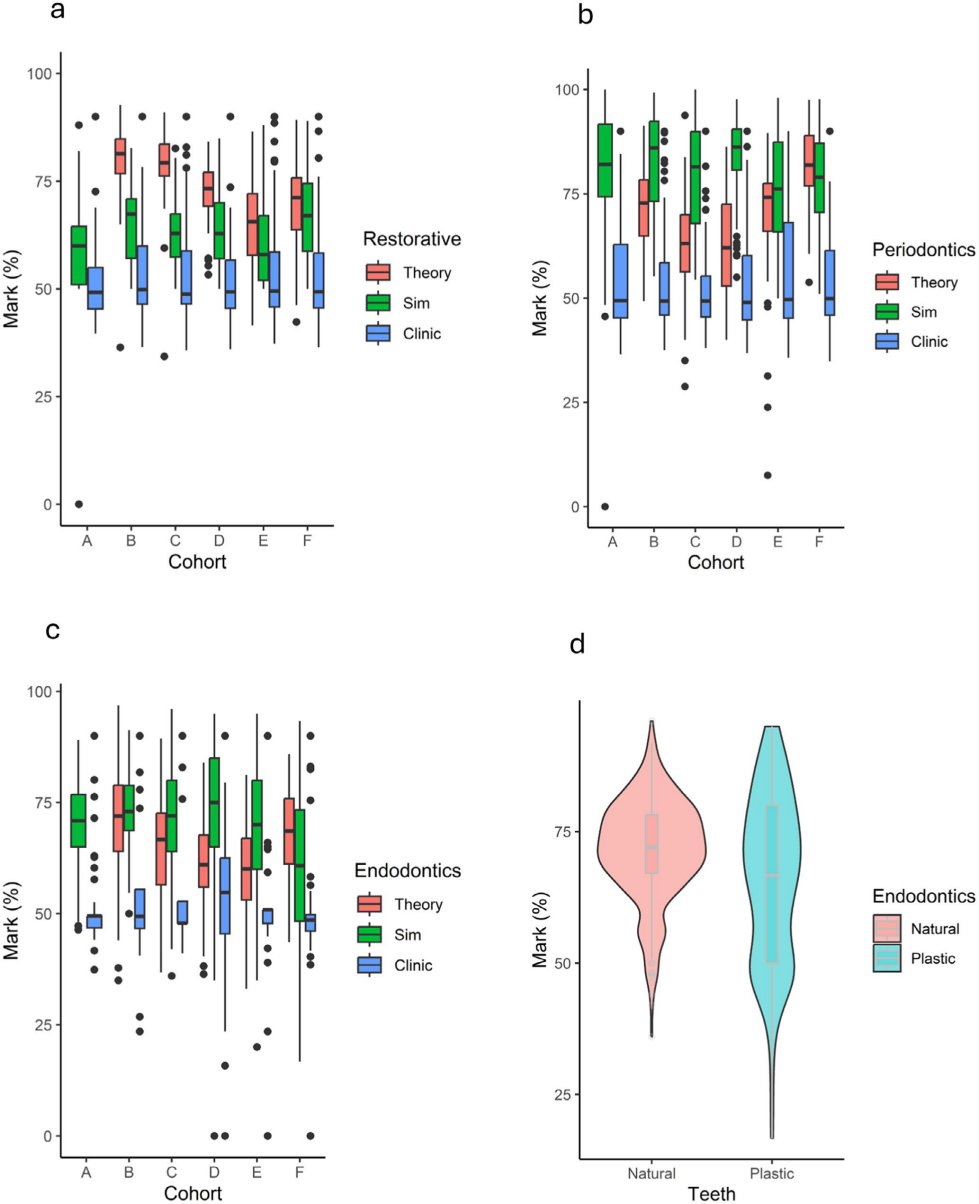
Box plot showing distribution of simulation and clinic marks as a percentage for each discipline a: Restorative dentistry (Restorative), b: Periodontics and c: Endodontics, and violin plot showing d: Distribution of Endodontic marks in simulation clinics using plastic and extracted teeth. Centre line of the boxplot represents the median; the minima and maxima of the box length correspond to the first and third quartiles (the 25th and 75th percentiles), whiskers extend from the box to no further than 1.5 * IQR (interquartile range) from the hinge. Data beyond the end of the whiskers are plotted individually.

The distribution of marks across all disciplines showed that students achieved higher marks in the pre‐clinical assessments (both theory and simulation) than in patient clinics for all six cohorts (Figure 2a–c). However, correlation analysis identified only two significant predictors of clinical performance: (i) pre‐clinical theory marks in DMD 2 were a weak but significant predictor of clinical marks in all three disciplines and (ii) a significant positive correlation between simulation and clinical marks was only found in Restorative. The results are summarised in Table 3.

**TABLE 3:** Results of correlation analysis between summative pre‐clinical assessments (theory and simulation) and clinical performance by discipline.

Discipline	Theory and Clinic *r* ^2^ (*p*)^a^	Simulation and Clinic *r* ^2^ (*p*)
**Periodontics**	**0.13** (0.01)	0.043 (0.35)
**Restorative dentistry**	**0.15** (0.00)	**0.15** (0.00)
**Endodontics**	**0.17** (0.00)	
Natural teeth		0.038 (0.53)
Plastic teeth		−0.014 (0.87)

*Note:* Significant results in bold.

^a^ Pearson's correlation coefficient (*r* ^2^) represents the direction and strength of the linear relationship.

A subset analysis was undertaken for Endo as the type of teeth used in simulation summative assessments transitioned from extracted teeth (cohorts A–D) to plastic teeth (cohorts E‐F). Figure 2d shows the distribution of marks by tooth type with a significant difference between assessments using extracted versus plastic teeth (*p* = 4.361 × 10^−5^, Welch *t*‐Test). Extracted teeth may potentially be a better predictor of clinical performance than plastic teeth, as a positive correlation was seen with the clinical marks (*r* ^2^ = 0.53, Table 3), however this association was not statistically significant.

## 4 Discussion

The results of this study initially appear to confirm the current consensus that pre‐clinical performance is not a good predictor of clinical performance. However, as this is a longitudinal, retrospective cohort analysis, our results can provide a more nuanced understanding of the impact of pre‐clinical assessments.

We found a significant, weak (*r* ^2^ scores less than 0.2) positive correlation between pre‐clinical theory and clinical assessments in all three disciplines which was consistent with previous studies [9, 12, 21, 22]. As our results are longitudinal, this may potentially indicate that the acquisition of knowledge in a pre‐clinical year (DMD 2) has some positive longer‐term impact in patient clinics where the knowledge is applied. This supports the timeless teaching paradigm that a strong theoretical understanding of a clinical subject supports clinical competency [23].

The correlation between simulation and clinical assessments is more complex. The only significant positive correlation we found was between simulation and clinical performance in Restorative. Dental programs focus on restorative dentistry training (greatest percentage of sessions in simulation and patient clinics) which reflects the clinical focus of general practice. From a pedagogical perspective, we attribute this result to the principles of behaviourism (repetitive learning) and constructivism [24]. Based on this conclusion, we would expect a similar result for Perio, which generally comes a close second in overall hours dedicated to a discipline within a dental program. However, this was not the case in this study, where no correlation was found between simulation and clinical performance in Perio, despite similarities in training with Restorative in terms of the length of the simulation program and content, which focuses on ergonomics, treatment techniques and patient safety from the aspect of managing instruments safely. Moreover, students undertake Perio procedures in both general practice clinics and specialist Periodontics clinics, which means they are consistently practising their skills and are continuously assessed by general educators and specialists. This suggests the quantity and frequency of simulation and clinical sessions may not be a good predictor of clinical performance. Considering this, the acquisition of technical skills on simulated teeth in Perio may benefit from the introduction of virtual simulation systems, which could assist students in integrating theoretical and practical skills, thereby enabling a smoother transition from pre‐clinical training to clinical practice and potentially enhancing clinical performance [25, 26].

In this study, performance in Endo simulation was not significantly correlated to performance in patient clinics. This finding was undoubtedly influenced by smaller sample sizes and the limited number of data points collected from the Endo procedures compared to the other disciplines. The reduced sample size was exacerbated by the exclusion of students who did not complete their Endo requirements. The inability to complete this clinical requirement may be attributed to difficulty in finding suitable cases, higher patient anxiety associated with pain and RCT procedures [27] and patient retention (multiple, long appointments compared to single, often shorter, appointments for scaling or a restoration).

Summative simulation assessments in Endo were undertaken on extracted teeth until 2017. Therefore, a subset analysis was done to investigate if tooth type was a factor in prognostication of clinical performance. Our results showed the simulation performance on extracted teeth has a weak positive correlation with clinical assessment; however, this was not significant. The results for plastic teeth were inconclusive. A previous study investigating the effect of tooth type evaluated the final radiographic appearance of an RCT completed in patients by students who had simulation training in extracted or plastic teeth and found tooth type did not influence clinical outcomes [14]. As a qualitative measure was used, it is difficult to draw comparisons with our findings. Further research is required to understand the influence of tooth type.

A notable advantage of this study is that it captures real world data. The data analysed is representative of assessment processes utilised by most Australian dental schools. Therefore, these results can inform practical changes to assessment compared to experimental studies where every effort is made to create ideal conditions. Study designs can account for sample sizes (with power calculations), selection of participants which may not reflect the range of abilities in an actual teaching environment, and most importantly the elimination of the need to calibrate. This is done using one to two assessors who mark all assessments. These conditions are unlikely to be reproducible in the typical teaching environment.

Real world data has its limitations, and an ongoing issue in dental education is the calibration of teaching staff. While the focus of the process is to ensure the extent of agreement between educators when evaluating a student's performance, it also includes the ability of an individual educator to consistently evaluate students over time [28]. Therefore, the data from this study reflect calibration processes involving multiple educators from different disciplines and the changing teaching paradigms of an individual educator as they gain teaching and clinical experience. Despite the sustained effort made to standardise assessment, there were statistically significant differences in marks between the cohorts across all disciplines except for Endo when using extracted teeth in simulation. There was no significant difference in the Endo delta score (difference between simulation and clinical marks) between cohorts A to D (*p* = 0.11 one way ANOVA). This result suggests that the calibration of assessors may have been influenced by tooth type, specifically issues with the typodonts when plastic teeth were first introduced in 2018. While this variation in marking is a limitation of this study, it reiterates the fundamental and ongoing struggle all dental schools face with assessment and standardisation. It is well documented in the current literature that calibration in dental education is notoriously difficult, mainly due to variations in teaching and clinical philosophy/attitudes of individual educators [28]. It also reveals the shortcomings of defining clinical competency using quantitative metrics like pre‐clinical summative assessment scores.

The ongoing interest in understanding the influence of pre‐clinical performance is crucial, as it addresses a fundamental aspect of dental education—clinical readiness. This includes the transition from plastic models (pre‐clinical) to real patients (clinical) during training and, ultimately, from student to graduate, reflecting preparedness for practice. Clinical readiness has proved more challenging to define compared to practice preparedness in the dental context [29, 30, 31, 32]. Numerous health professions have attempted to define clinical readiness; however, it remains poorly understood in the dental literature. To provide a dental context through modification of existing definitions, clinical readiness can be described as (i) ‘Knowing’ which encompasses theoretical knowledge and synthesis of prior learning and (ii) ‘Being’ which includes psychomotor skills, clinical decision making (resilience and adaptability) and professionalism [33]. This definition clearly indicates that a constellation of qualitative and quantitative metrics is required to better assess this important attribute and provide a more wholistic profile of a student technician as they transition to clinician.

Future technological advances in simulation training will result in the eventual replacement of plastic teeth with multimedia supported simulators, virtual reality, artificial intelligence and more sophisticated systems in simulation training [34]. Significant changes are expected in written assessments as Artificial Intelligence increasingly disrupts traditional assessments such as SBA and SAQs [35]. These new technologies may increase the prognostic value of simulation assessment results for clinical performance. However, it is unlikely that we can solely rely on pre‐clinical summative assessment performance to assess clinical readiness.

## 5 Conclusions

Academic progression in dentistry remains reliant on the results of theory and simulation summative assessments in the pre‐clinical program to determine student readiness for patient clinics. The results of this study suggest that pre‐clinical theory performance may potentially predict clinical performance in Restorative, Perio and Endo, while simulation competency may partially predict clinical performance in Restorative, but not in Perio or Endo. The results of our study suggest a new approach is required to understand and define clinical readiness, looking at a broader range of measures including communication and professionalism.

## Author Contributions

Y.A. conceived the idea with T.D.‐R., F.E.M., A.S., B.C.G. and S.S. Data collection and curation was coordinated by B.C.G. All authors interpreted the data prior to statistical analysis by S.S. Data visualisation was done by S.S. and Y.A. Y.A., T.D.‐R., F.E.M. and S.S. co‐wrote the paper with support from A.S. All authors read and approved the final manuscript.

## Ethics Statement

This was a retrospective cohort study using anonymised assessment data, therefore student consent to participate was not required. The University of Sydney Human Ethics Committee waived the need for informed consent. The study methodology adhered to the relevant guidelines set out in the Australian National Statement on Ethical Conduct in Human Research (2018). Ethics approval for this study was obtained through the University of Sydney Human Ethics Committee (2021/337).

## Conflicts of Interest

The authors declare no conflicts of interest.

## Supporting information


Tables S1–S6.

## Data Availability

All data generated or analysed during this study are included in this published article as Tables S1–S6.
